# Prevention of hepatocellular cancer: one of the most cost-effective ways to reduce adult mortality?

**DOI:** 10.1038/sj.bjc.6690813

**Published:** 1999-12

**Authors:** A J Hall, P G Smith

**Affiliations:** Department of Infectious and Tropical Diseases, London School of Hygiene & Tropical Medicine, Keppel Street, London WC1E 7HT, UK


British Journal of Cancer (1999) 81(7), 1097–1098
© 1999 Cancer Research Campaign
Article no. bjoc.1999.0813

Editorial

### Prevention of hepatocellular cancer: one of the most cost-effective ways to reduce adult mortality?

**AJ Hall and PG Smith**

Department of Infectious and Tropical Diseases, London School of Hygiene & Tropical Medicine, Keppel Street, London WC1E 7HT, UK

In 1965, Blumberg, Alter and Visnich described a 'new' antigen in sera from patients with leukaemia, and in 1970, Prince and colleagues went on to show that this SH (serum hepatitis) antigen was associated with chronic hepatitis. A decade later, Beasley et al (1981), working in Taiwan, showed that the risk of developing liver cancer among men positive for this marker of persistent hepatitis B virus (HBV) infection was approximately 200 times greater than that among men without the marker. Although this relative risk fell with further follow-up to around 100 (Beasley and Hwang, 1991), the very strong association between the hepatitis B carrier state and the risk of hepatocellular cancer has been replicated in multiple case–control and cohort studies in various parts of the world (IARC, 1994). The strength and consistency of these findings, together with evidence of biological plausibility gained from animal models, led the International Agency for Research on Cancer to conclude that there is sufficient evidence for the carcinogenicity of chronic infection with HBV in humans (IARC, 1994).

It has been estimated that at least 60% of cases of primary liver cancer worldwide and 67% in developing countries are attributable to persistent infection with HBV (Pisani et al, 1997). The burden of mortality due to chronic hepatitis and cirrhosis that is attributable to hepatitis B infection is much less clear due to a paucity of relevant studies but is likely to exceed that due to liver cancer. It has been estimated that around 40% of hepatitis B carriers will die as a result of the infection. Since approximately 20% of adults in China and sub-Saharan Africa are carriers, this signifies a major global public health problem.

Only a minority of those infected with HBV become persistently infected, the major determinant being the age at first exposure to the virus (Edmunds et al, 1993). In China, where infection occurring around the time of birth from an infectious mother is common, about 40% of carriers are infected perinatally (IARC, 1994). There, about 90% of children born to carrier mothers are infected and perinatal infection is associated with about a 90% risk of becoming a carrier (Beasley et al, 1977; Mitusda et al, 1989). In many other parts of Asia and in sub-Saharan Africa, infection is most common in childhood. In The Gambia, for example, between 35% and 70% of children were found to have been infected by the age of 5 years (Whittle et al, 1990). Although the prevalence of infectious mothers in such populations is substantially lower than in China, the rates of chronic infection in the population are still high, as the probability of becoming a carrier following infection in early childhood is around 20–30%. In contrast, the risk of becoming a carrier following infection in adolescence or adult life is less than 10%. This pattern makes the options for prevention clear. Either primary prevention of persistent infection must be undertaken early in life (particularly very early in life in China), or secondary prevention measures must be directed at reducing the risk associated with carriage later in life.

Progress on these two possible intervention strategies has been unequal. Secondary prevention by modification of carriage can now be made by anti-viral therapy but is currently expensive and its long-term effectiveness has yet to be demonstrated. The high cost is a particularly important issue since the vast majority of carriers in the world live in Asia and Africa, where resources are very limited. Alcohol avoidance is likely to reduce the risks associated with carriage but this is a difficult intervention to implement. The likely role of dietary aflatoxin has become clearer in recent years. Cohort studies in which stored samples have been analysed for biomarkers of aflatoxin exposure on a case–control basis indicate that aflatoxin exposure increases the risk of liver cancer by around twofold (Ross et al, 1992). However, because the risk appears to be multiplicative, the impact of such exposure among hepatitis B carriers, who are at high risk of liver cancer, may be substantial. This has led to more recent focus on the possibility of benefiting carriers by reducing their aflatoxin exposure. Oltipraz, an anti-schistosomal drug, induces aflatoxin metabolizing enzymes and has been shown to inhibit aflatoxin-induced hepatocarcinogenesis in animal models (Kensler et al, 1999). Whilst some trials of this chemotherapeutic agent in humans have been conducted (Wang et al, 1999), this does not represent a realistic option in the affected resource-poor countries. A more attractive agricultural approach is to reduce contamination of locally consumed foodstuff; however, field trials are needed using human biomarker exposure as the end point to assess their effectiveness, and ultimately intervention studies to assess whether simple, technologically appropriate interventions can reduce the incidence of hepatocellular carcinoma.

In contrast, great strides have been made in primary prevention. Hepatitis B vaccine has been available since 1980. Initial studies in the USA showed its effectiveness in preventing infection and acute hepatitis. More importantly, studies in China, Taiwan and West Africa demonstrated that vaccination at birth or in early childhood prevented the chronic carrier state: the efficacy in China was between 70% and 85% (Sun et al, 1986, 1991) but in Africa where perinatal transmission is less common it was over 90% (Fortuin et al, 1993). Recently, this protection has been shown to persist up to 9 years of age (Viviani et al, 1999), which is more

*Received 19 April 1999*
*Revised 1 May 1999*
*Accepted 18 May 1999*

*Correspondence to:* PG Smith important since it covers the period of life when the risk of carriage if infected is high. Thus, even if vaccine-induced immunity wanes later in life, there is good reason to suppose that carriage will have been prevented in a substantial proportion of vaccinated individuals, even without any booster vaccination. Direct evidence of the protective effect of hepatitis B vaccination against liver cancer comes from a study in Taiwan where all newborns have been vaccinated since the mid 1980s. The rate of liver cancer among children aged 6–9 years in the vaccinated cohort was 1.3 million$^{-1}$ year$^{-1}$ compared to a rate of 5.2 million$^{-1}$ year$^{-1}$ in the preceding unvaccinated cohort (Chang et al, 1997). Whilst these data have accumulated the price of vaccine has fallen dramatically from around $40 per dose in the early 1980s to less than $1 per dose now. At this price it has been estimated that, in West Africa, the prevention of one carrier of the virus costs between $20 and $30, and the prevention of a case of primary liver cancer (which is uniformly fatal) only $130 (Hall et al, 1993). Since the target for vaccination is infants this is a once and for all lifetime cost for each individual in terms of prevention of HBV carriage and liver cancer. The corresponding cost in measles vaccination is around $40 per death prevented, and that of preventing a diarrhoeal death by improved water supply and sanitation is estimated at $1200 per death prevented.

This evidence led the WHO to recommend universal hepatitis B vaccination for all countries of the world either in infancy (where childhood infection is common) or in pre-adolescence (where adolescent or adult infection is the main problem). Currently, although more than 100 countries have instituted this policy, there is a marked geographical disparity. The African continent lags far behind the rest of the world, despite the public health importance of liver cancer there, with only two countries having integrated hepatitis B vaccine into their national childhood vaccine programmes. The limited cancer registry data in sub-Saharan Africa suggests that this is the commonest cancer in men and the second commonest in women (Bah et al, 1990). It is estimated that some 10% of adult West Africans will die prematurely as a result of infection with the virus.

Most causes of adult mortality have proved intractable to simple preventive interventions. Many of the potential preventive interventions require difficult to implement changes in lifestyle. Given the importance of the long-term sequelae of hepatitis B in Africa and other developing regions of the world, the prevention of the hepatitis B carrier state by the introduction of hepatitis B vaccination into routine universal childhood vaccination programmes is probably one of the simplest and most cost-effective means of reducing levels of adult mortality. Indeed, it is difficult to think of any other intervention which costs less than $3 per individual and which could have such a significant impact on adult mortality in developing countries. It is true that the major benefit would not be felt for several decades after such vaccination started but it would seem very short-sighted not to give this preventive measure high priority now. Most welcome, therefore, is the recent announcement that the expansion of programmes of hepatitis B vaccination in developing countries is to be given priority by the Programme for Appropriate Technology in Health (PATH) with substantial financial support from the Bill and Melinda Gates Children's Vaccine Program.

### REFERENCES

Bah E, Hall AJ and Inskip HM (1990) The first two years of the Gambian National Cancer Registry. *Br J Cancer* **62**: 647–650

Beasley RP, Trepo C, Stevens CE and Szmuness W (1977) The e antigen and vertical transmission of hepatitis B surface antigen. *Am J Epidemiol* **105**: 94–98

Beasley RP and Hwang LY (1991) Overview on the epidemiology of hepatocellular carcinoma. In: *Viral Hepatitis and Liver Disease*, Hollinger FB, Lemon SM and Margolis HS (eds), pp. 532–535. Williams & Wilkins: Baltimore.

Beasley RP, Hwang LY, Lin CC and Chien CS (1981) Hepatocellular carcinoma and hepatitis B virus. A prospective study of 22 707 men in Taiwan. *Lancet* **ii**: 1129–1133

Blumberg BS, Alter HJ and Visnich S (1965) A 'new' antigen in leukemia sera. *J Am Med Assoc* **191**: 101–106

Chang MH, Chen CJ, Lai MS, Hsu HM, Wu TC, Kong MS, Liang DC, Shau WY and Chen DS (1997) Universal hepatitis B vaccination in Taiwan and the incidence of hepatocellular carcinoma in children. *N Engl J Med* **336**: 1855–1859

Edmunds WJ, Medley GF, Nokes DJ, Hall AJ and Whittle HC (1993) The influence of age on the development of the hepatitis B carrier state. *Proc R Soc Lond B* **253**: 197–201

Fortuin M, Chotard J, Jack A, Maine N, Mendy M, Hall AJ, Inskip H, George M and Whittle H (1993) Efficacy of hepatitis B vaccine in The Gambian Expanded Programme of Immunisation. *Lancet* **341**: 1129–1131

Hall AJ, Robertson RL, Crivelli PE, Lowe Y, Inskip H, Snow SK and Whittle H (1993) Cost-effectiveness of hepatitis B vaccine in The Gambia. *Trans Roy Soc Trop Med Hyg* **87**: 333–336

IARC (1994) IARC monograph on the evaluation of carcinogenic risks to humans. Volume 59. *Hepatitis Viruses*. International Agency for Research on Cancer: Lyon, France.

Kensler TW, Groopman JD, Sutter TR, Curphey TJ and Roebuck BD (1999) Development of cancer chemotherapeutic agents: oltipraz as a paradigm. *Chem Res Toxicol* **12**: 113–126

Mitsuda T, Mori T, Ookawa N, Aihara Y, Kosuge K, Yokota S, Ibe M, Shimizu H, Yoshida N and Matsuyama S (1989) Demonstration of mother-to-infant transmission of hepatitis B virus by means of polymerase chain reaction. *Lancet* **ii**: 886–888

Pisani P, Parkin MP, Munoz N and Ferlay J (1997) Cancer and infection: estimates of the attributable fraction in 1990. *Cancer Epidemiol Biomarkers Prev* **6**: 387–400

Prince AM, Leblanc L, Krohn K, Masseyeff R and Alpert ME (1970) SH antigen and chronic liver disease. *Lancet* **ii**: 717–718

Ross RK, Yuan J-M, Yu MC, Wogan GN, Qian G-S, Tu J-T, Groopman JD, Gao Y-T and Henderson BE (1992) Urinary aflatoxin biomarkers and risk of hepatocellular carcinoma. *Lancet* **339**: 943–946

Sun TT, Chu YR, Ni ZQ, Lu JH, Huang F, Ni ZP, Pei XF, Yu ZI and Liu GT (1986) A pilot study on universal immunization of newborn infants in an area of hepatitis B and primary hepatocellular carcinoma prevalence with a low dose of hepatitis B vaccine. *J Cell Physiol* **4**: 83–90

Sun Z, Zhu Y, Stjernsward J, Hilleman M, Collins R, Zhen Y, Hsia CC, Lu J, Huang F, Ni Z et al (1991) Design and compliance of HBV vaccination trial on newborns to prevent hepatocellular carcinoma and 5-year results of its pilot study. *Cancer Detect Prev* **15**: 313–318

Viviani S, Jack A, Hall AJ, Maine N, Mendy M, Montesano R and Whittle HC (1999) Hepatitis B vaccine efficacy at nine years of age following infant vaccination in The Gambia. *Vaccine* **17**: 2946–2950

Wang JS, Shen X, He X, Zhu YR, Zhang BC, Wang JB, Qian GS, Kuang SY, Zarba A, Egner PA, Jacobson LP, Munoz A, Helzlsouer KJ, Groopman JD and Kensler TW (1999) Protective alterations in phase 1 and 2 metabolism of aflatoxin B1 by oltipraz in residents of Qidong, People's Republic of China. *J Natl Cancer Inst* **91**: 347–354

Whittle H, Inskip H, Bradley AK, McLaughlan K, Shenton F, Lamb W, Eccles J, Baker BA and Hall AJ (1990) The pattern of childhood hepatitis B infection in two Gambian villages. *J Infect Dis* **161**: 1112–1115

*© 1999 Cancer Research Campaign*

